# The Effect of the Type and Concentration of Garlic (*Allium sativum*) on Heinz Body Concentrations in Canine Erythrocytes—An In Vitro Study

**DOI:** 10.3390/ani15213188

**Published:** 2025-11-01

**Authors:** Klaudia Beleć, Justyna Barć, Olga Lasek

**Affiliations:** Department of Animal Nutrition and Fisheries, University of Agriculture in Krakow, Al. Mickiewicza 24/28, 30-059 Krakow, Poland; klaudia.belec@urk.edu.pl (K.B.); olga.lasek@urk.edu.pl (O.L.)

**Keywords:** garlic, canine nutrition, hemolysis, Heinz bodies, hemoglobin

## Abstract

**Simple Summary:**

Garlic is a popular food ingredient and natural remedy for people, but its use in dogs may not be safe. Many owners believe that garlic can protect their pets against parasites or improve their health, yet garlic also contains substances that may damage red blood cells. When red blood cells are harmed, they lose their ability to carry oxygen, which can lead to anemia. In this study, we wanted to find out whether fresh, dried, and granulated garlic can cause different levels of damage to dog red blood cells. We mixed garlic extracts with blood samples taken from healthy dogs during routine veterinary procedures. We then measured the amount of hemoglobin, the protein that carries oxygen, and looked at the cells under a microscope to check for signs of damage. We found that all forms of garlic caused some harm to the red blood cells, but dried and granulated garlic were more damaging than fresh garlic. These results suggest that processing garlic may make it more harmful for dogs. Our findings highlight the need for caution when using garlic in pet diets and show that more research is needed to identify what level, if any, of garlic is safe in dog food and supplements.

**Abstract:**

Garlic (*Allium sativum*) is widely used in human diets and medicine, but its safety for dogs remains uncertain. Heinz bodies in red blood cells are indicators of oxidative damage, which may lead to hemolytic anemia. This study evaluated the effects of different forms and concentrations of garlic on canine erythrocytes in vitro. The experiment consisted of two phases: Phase 1 compared fresh, dried, and granulated garlic, while Phase 2 assessed two concentrations of granulated garlic (0.1 and 0.2 g/mL). Blood from healthy dogs was incubated with ethanol extracts of garlic. Hemolysis was measured spectrophotometrically by hemoglobin release, and blood smears were examined for Heinz bodies and eccentrocytes. All garlic preparations caused hemolysis, most strongly granulated garlic at 0.2 g/mL (*p* < 0.01). Blood smears confirmed red blood cell damage, with more Heinz bodies and eccentrocytes in dried and granulated garlic samples. In conclusion, the form and concentration of garlic strongly influence its hemolytic activity. Processing methods such as drying and granulation may enhance the release of reactive compounds, increasing the risk of oxidative damage to canine red blood cells.

## 1. Introduction

Garlic (*Allium sativum*) is one of the oldest cultivated spices and medicinal plants used in human nutrition. Due to its health-promoting properties, including antibacterial, antiparasitic, antioxidant and immunostimulatory effects, it has found wide application not only in culinary practices but also in medicine, enhancing the body’s overall resistance [1,2,3]. For this reason, garlic is widely used in the human diet, and its popularity has also led to attempts to utilize it in animal nutrition [4,5]. The feed additive market offers numerous garlic-containing products for dogs and cats, available in various forms such as granules, powders, and extracts. Manufacturers typically highlight garlic’s purported health-promoting properties, including benefits for the digestive system, immunity, and protection against parasites. However, information regarding the potential toxicity of this ingredient is often omitted, and the garlic doses included in the preparations are not precisely specified (Table 1).

There is, however, evidence confirming the toxic effects of garlic in certain animal species, including sheep [1], horses [6], and dogs [7]. Garlic toxicity is associated with the presence of specific organosulfur compounds (i.e., thiosulfates, sulfides), as well as potentially harmful sulfur-containing esters [1,8].

These compounds induce oxidative damage to erythrocyte membranes and cause denaturation of hemoglobin. Due to oxidative action, hemoglobin is converted into methemoglobin–a form incapable of transporting oxygen because of the oxidation of Fe^2+^ to Fe^3+^. As a result, As a result, Heinz bodies (inclusions formed by the denaturation and precipitation of hemoglobin attached to the erythrocyte membrane) and eccentrocytes (red blood cells with eccentrically located hemoglobin caused by oxidative membrane damage) are formed and rapidly removed from circulation, resulting in hemoglobinemia and hemoglobinuria. The severity of oxidative damage, including the formation of Heinz bodies and eccentrocytes, is dose-dependent and species-specific due to differences in erythrocyte metabolism and antioxidant enzyme activity [9,10,11,12].

According to the annual report of the Veterinary Poison Information Service (VPIS) [13], plants of the genus *Allium* were the fourth most frequently reported cause of poisoning in the animals covered by the report. In dogs, however, onion (*Allium cepa*) toxicity was reported more often (0.9% of all reported cases), whereas in cats, *Allium* species were the most common toxic factor (3.3% of all reported cases). It has been demonstrated that, compared to humans, canine erythrocytes contain lower levels and exhibit reduced activity of the enzyme catalase [14]. Catalase is an enzyme with antioxidant activity in cells. Its decreased abundance and activity may impair the organism’s ability to cope with strongly oxidizing substances, including the sulfur compounds present in garlic.

Clinical signs of garlic poisoning include gastrointestinal, hematological, and systemic signs (e.g., lethargy, vomiting, anemia, jaundice) [12,15,16]. The treatment of garlic poisoning is nonspecific and primarily symptomatic. There is no known antidote, and supportive therapy with antioxidants provides only limited benefits. In more severe cases, blood transfusions and oxygen therapy may be necessary [16,17,18].

According to European Pet Food Industry Federation (FEDIAF) [19], reported toxic effects in dogs were observed after administration of 1.25 mL of garlic extract per kg body weight, which corresponds to approximately 5 g/kg body weight of fresh garlic given daily for seven days. This dose is comparable to amounts reported in cases of onion poisoning. Similarly, the National Research Council (NRC) [20] defines potential safe intake of garlic with reference to fresh garlic. Although garlic is commonly included in commercial feed additives, data on its safe dosage for companion animals are inconsistent, and the actual concentrations used in these products are often undisclosed. This creates a significant knowledge gap regarding the balance between potential health benefits and risks. We hypothesized that garlic, depending on its form and concentration, may exert a dose-dependent oxidative effect on canine erythrocytes, leading to the formation of Heinz bodies and other hemolytic changes. To address this issue, the aim of the present study was to determine, under in vitro conditions, the effects of extracts from different forms of garlic and concentrations of granulated garlic on erythrocytes isolated from canine blood.

## 2. Materials and Methods

### 2.1. Experimental Design

The experiment was conducted in two phases to evaluate the potential harmful effects of garlic on canine erythrocytes. Both phases followed a uniform analytical methodology but differed in scope and in the number and composition of the treatment groups. In the first phase, the effects of different garlic forms (fresh, dried, and granulated) were compared, whereas in the second phase, the impact of increasing concentrations of granulated garlic was evaluated.

### 2.2. Garlic Extract Preparation

In the first phase of the experiment, the effects of three different forms of garlic were compared: fresh, dried, and granulated. Fresh garlic (peeled and finely chopped) and dried garlic (from laboratory stocks) were extracted alongside a commercial granulated preparation marketed as a feed additive for dogs and cats. These forms were chosen because they are readily accessible to pet owners–some are available as feed additives, while others are commonly recommended in online sources as potential supplements for dogs. Thus, all represent forms of garlic that may realistically be ingested by companion animals, either accidentally or through intentional administration.

The second phase of the experiment focused on two different concentrations of granulated garlic extract, prepared from the same commercial product, in order to assess dose-dependent effects on erythrocytes.

Garlic extracts were prepared according to the method of Hu et al. [1], with modifications. Extraction was performed in 70% ethanol (ρ = 0.88 g/cm^3^; T913, Carl Roth GmbH, Karlsruhe, Germany) using proportions standardized to dry weight: 0.25 g/mL for fresh garlic and 0.11 g/mL for dried and granulated garlic. Ethanol was chosen as the extraction solvent because 70% ethanol efficiently solubilizes both polar and non-polar sulfur compounds, including allicin and its derivatives, while minimizing their degradation. This approach also reflects commonly used extraction conditions in phytochemical and toxicological studies of garlic. For the second experiment, two granulated garlic extracts of different concentrations were prepared: 0.10 g/mL and 0.20 g/mL in 70% ethanol. All extracts were subsequently diluted with phosphate-buffered saline (PBS) at a ratio of 1:88 prior to incubation, in order to minimize the effect of ethanol on the cells. A vehicle control (PBS with ethanol at the same final concentration) was included.

### 2.3. Collection of Blood Samples

For both phases of the experiment, blood was collected from five healthy mixed-breed male dogs, aged 10–14 months and weighing 15–20 kg. Samples were obtained during routine pre-castration clinical procedures, with the owners’ informed consent. Blood was collected into tubes containing EDTA as an anticoagulant, and the remaining material, left after standard hematological and biochemical analyses, was used for the in vitro experiment. No a priori sample size calculation was performed; the sample size was constrained by ethical and logistical considerations for surplus clinical material and is in line with similar in vitro erythrocyte studies. Inclusion criteria were clinically healthy status, confirmed by both physical examination and recent hematological testing performed as part of standard pre-castration procedures, as well as owner consent. Dogs showing anemia or any erythrocyte abnormalities were considered ineligible for inclusion. No animals, experimental units, or data points were excluded from analysis.

### 2.4. Erythrocyte Isolation and Incubation

Erythrocytes were isolated following the method of Hanson et al. [21]. Whole blood was centrifuged three times at 4 °C (2000× *g*, Sorvall ST16R, Thermo Scientific, Waltham, MA, USA), and the erythrocyte pellet was washed each time with PBS. After isolation, incubation mixtures were prepared by combining erythrocytes with the appropriately diluted garlic extracts or with the vehicle control. In the first phase of the experiment, the treatment groups included: control (PBS with ethanol), fresh garlic extract, dried garlic extract, and granulated garlic extract. In the second phase of the experiment, erythrocytes were incubated with control (PBS with ethanol), granulated garlic extract at 0.10 g/mL, or granulated garlic extract at 0.20 g/mL. All samples were incubated in a water bath (Elpin-Plus 357, ELPIN, Mińsk Mazowiecki, Poland) at 37 °C for one hour. The experimental unit was the erythrocyte suspension obtained from a single donor dog. For each dog and treatment, measurements were performed in triplicate and averaged to one value per dog per treatment.

### 2.5. Measurement of Hemoglobin Concentration and Heinz Body Counts

After incubation, hemoglobin concentration was determined using the cyanmethemoglobin method [22] with an ATI Unicam 8675 colorimeter (ATI Unicam, Cambridge, UK). Absorbance was measured 15 and 25 min after the addition of Drabkin’s reagent. Hemoglobin concentration (g/100 mL) was calculated using the standard coefficient (16.44) and correcting for sample dilution.

The number of Heinz bodies was assessed microscopically according to Dacie and Lewis [23]. Stained erythrocytes containing Heinz bodies were enumerated using a Delta Optical Genetic Pro light microscope (Delta Optical, Nowe Osiny, Poland).

### 2.6. Statistical Analysis

Data from the experiment were analyzed using STATISTICA 12 software [24]. Normality of distribution (Shapiro–Wilk test) and homogeneity of variance (Levene’s test) were verified before applying parametric tests. Group means were compared using one-way analysis of variance (ANOVA) followed by Tukey’s post hoc test. The relationship between hemoglobin concentration and Heinz body counts was examined using Pearson’s correlation coefficient. Results were considered statistically significant at *p* < 0.05. All measurements were performed in triplicate.

## 3. Results

### 3.1. Heinz Body Formation

**Figure 1:**
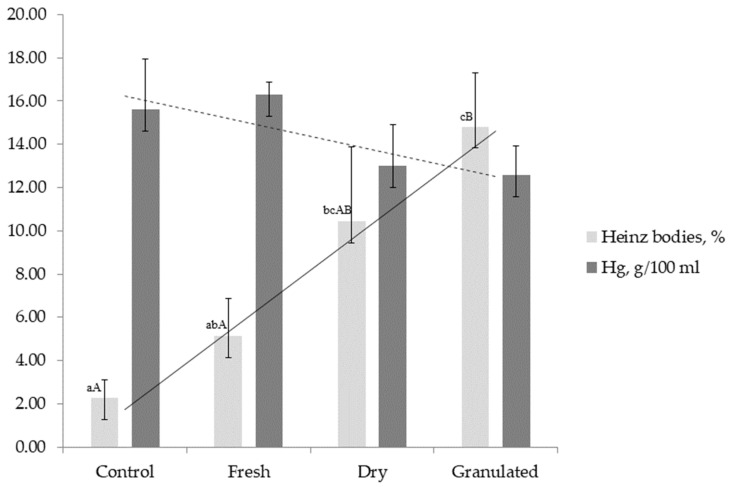
Hemoglobin concentration (Hg, g/100 mL; dark bars) and proportion of Heinz bodies (%; light bars) in canine erythrocytes incubated with extracts of fresh, dried, and granulated garlic compared with the control group (PBS). Data are presented as mean ± SD. Different superscript letters indicate statistically significant differences between groups (lowercase letters: *p* < 0.05; uppercase letters: *p* < 0.01). A negative correlation (r = −0.70) was observed between hemoglobin concentration and the proportion of Heinz bodies across treatments.

Incubation of canine erythrocytes with garlic extracts resulted in a dose- and form-dependent increase in Heinz body formation. Among the three forms tested, the granulated garlic extract produced the strongest effect, with approximately 15.0 ± 2.5% of erythrocytes containing Heinz bodies, compared with 5.0 ± 1.7% after incubation with fresh garlic extract (*p* < 0.01) and values close to control levels in the dried garlic group (Figure 1, Table 2).

The proportion of damaged cells after exposure to granulated garlic extract was nearly fivefold higher than in the PBS control (*p* < 0.01), whereas the increase observed for the fresh garlic extract was not statistically significant (*p* = 0.09).

**Figure 2:**
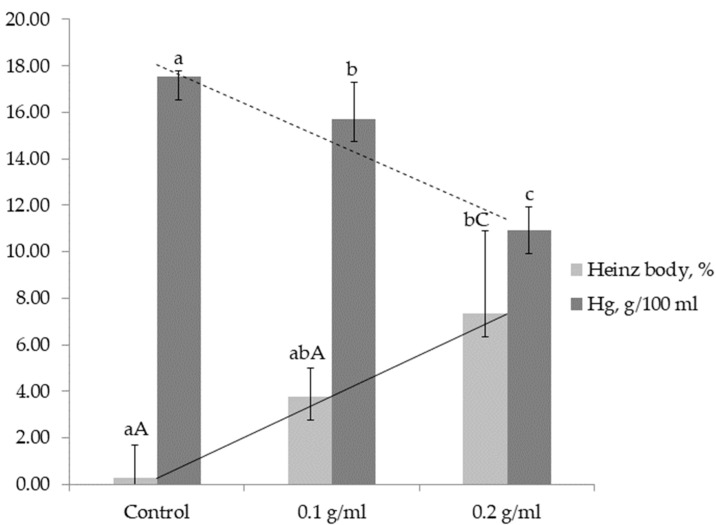
Hemoglobin concentration (Hb, g/100 mL; dark bars) and proportion of Heinz bodies (%; light bars) in canine erythrocytes incubated with granulated garlic extracts at concentrations of 0.1 g/mL and 0.2 g/mL compared with the control group (PBS). Data are presented as mean ± SD. Different superscript lowercase letters indicate statistically significant differences between groups at *p* < 0.05, while uppercase letters denote highly significant differences at *p* < 0.01. A clear dose-dependent effect was observed: increasing garlic concentration was associated with higher Heinz body formation and lower hemoglobin levels, demonstrating a strong negative correlation (r = −0.81) between these two parameters.

In the second part of the study, using different concentrations of granulated garlic, the extract at 0.20 g/mL caused Heinz body formation in 7.5 ± 1.0% of erythrocytes, whereas the 0.10 g/mL extract induced changes in 4.0 ± 1.6% of cells (Figure 2).

Control samples showed only 0.3 ± 0.3% Heinz bodies, which was ~25-fold and ~12-fold lower than the 0.20 g/mL and 0.10 g/mL treatments, respectively (Table 3).

### 3.2. Hemoglobin Concentration

Exposure to granulated garlic extract led to a reduction in hemoglobin concentration. Erythrocytes incubated with the granulated garlic extract exhibited hemoglobin levels that were on average 3 g/100 mL lower than in the control group, although this trend did not reach statistical significance (*p* = 0.09). Fresh and dried garlic extracts did not cause marked changes (Table 2).

In the concentration-dependent phase of the experiment, the greatest decrease was observed with the 0.20 g/mL extract, where hemoglobin concentration was 7 g/100 mL lower than in controls (*p* < 0.05). The 0.10 g/mL extract produced an intermediate effect (Table 3).

### 3.3. Correlation Analysis

Correlation analysis confirmed the relationship between hemoglobin concentration and the presence of Heinz bodies. Across treatments, a strong negative correlation was found between the proportion of Heinz body-containing erythrocytes and hemoglobin levels (r = −0.70 in the form comparison; r = −0.81 in the concentration experiment; both *p* < 0.05). Conversely, the proportion of intact erythrocytes correlated positively with hemoglobin concentration (r = 0.74 in the form comparison; r = 0.72 in the concentration experiment; both *p* < 0.05).

## 4. Discussion

The results of the present study confirm that garlic (*Allium sativum*) suggest potential toxic effects on canine erythrocytes, consistent with earlier reports [1,7,16]. The toxicity of garlic and other *Allium* species, such as onion, has been attributed to organosulfur compounds, including thiosulfates and diallyl derivatives, which cause oxidative damage to erythrocyte membranes and hemoglobin. This leads to the formation of Heinz bodies and eccentrocytes and may ultimately result in hemolytic anemia [12]. For example, Lee et al. [7] demonstrated that dogs receiving 5 g/kg body weight of fresh garlic daily for seven days developed significant hematological changes, including reduced hematocrit and hemoglobin concentration as well as the presence of Heinz bodies, although no clinical signs of anemia were observed. Importantly, this dose is very high and would require a 20 kg dog to consume approximately 100 g of fresh garlic (15–20 cloves) per day.

Our in vitro experiment demonstrated that the form of garlic significantly influences its hemolytic potential. Granulated and dried garlic caused a higher proportion of damaged erythrocytes and Heinz bodies than fresh garlic, which produced only mild changes. These findings suggest that technological processing may alter the chemical profile of garlic or increase the bioavailability of toxic constituents, thereby enhancing oxidative damage to red blood cells. Although the exact mechanisms remain unclear, it is known that thermal and technological processing can modify garlic’s biochemical properties [25,26]. For instance, alliinase–the enzyme responsible for the conversion of alliin to allicin–is heat-labile [27]. While allicin contributes to garlic’s odor and has been widely studied for its biological activities, other processing methods, such as low-temperature drying or lyophilization, can preserve some of the biochemical properties of fresh garlic [28].

Among processed forms, aged garlic extract (AGE) represents a unique case. AGE is produced by long-term soaking of garlic in aqueous ethanol and contains stable, water-soluble sulfur compounds with lower reactivity toward erythrocytes [29,30]. In Beagle dogs, long-term AGE administration (up to 90 mg/kg/day for 12 weeks) did not cause hematological or biochemical alterations or induce Heinz body formation, and even showed activation of the Nrf2 pathway and increased antioxidant enzyme expression [29,31]. However, AGE safety has been documented only for specific doses and durations, and its relevance to chronic use under household conditions remains uncertain. Moreover, most commercial animal supplements contain fresh, dried, or granulated garlic with poorly defined chemical composition, and manufacturers rarely provide precise information on the concentration of active compounds (Table 1).

In addition to hematotoxicity, garlic has been associated with gastrointestinal effects. Hoshito et al. [32] demonstrated that direct administration of raw or cooked garlic powders to the gastric mucosa caused lesions and erythema, whereas AGE did not. Although this experimental approach differs from typical dietary intake, it illustrates the potential for gastric irritation following supplementation.

Despite potential risks, garlic continues to be marketed for dogs, often with claims regarding antiparasitic, antimicrobial, cardiovascular, or immunostimulatory effects. However, most available studies supporting such claims are small, methodologically limited, and do not evaluate safety. For example, Orengo [33] suggested partial activity of garlic against *Ancylostoma caninum* eggs and larvae, but studies were based on small sample sizes or performed directly on fecal samples. Similarly, flea-control studies [34] reported reduced flea counts, but lacked systematic health monitoring, involved very few animals, or relied on external rather than oral application. Garlic has also been investigated for cardiovascular support, but results are inconsistent: while cooked garlic extracts reduced thrombus formation [35] and transiently lowered blood pressure [36], other studies found no significant cardiovascular effects [37]. Antimicrobial studies reported inhibition of *Helicobacter* spp. [38] or *Salmonella enteritidis* [39], but either lacked eradication efficacy, omitted hematological assessments, or were conducted in vitro with potentially toxic ethanol extracts. Collectively, these reports suggest that while garlic may display some biological activity, the evidence is inconsistent and often outweighed by concerns regarding safety.

Clinical reports further support the risk of garlic toxicity. Case studies describe dogs developing Heinz bodies, eccentrocytes, hemolytic anemia, or systemic clinical signs after ingesting roasted or raw garlic [11,40]. Epidemiological data indicate that *Allium* species are a frequent cause of poisoning in dogs and cats [13]. While onions are generally considered more hazardous [41], they are rarely used in pet diets, whereas garlic is more commonly included in supplements, increasing the likelihood of exposure. Breed predispositions have been reported, with Akita, Shiba, and Jindo dogs showing increased susceptibility due to erythrocyte characteristics [42]. Breed-specific sensitivity may reflect differences in erythrocyte membrane stability or antioxidant defences. However, toxic effects have also been observed in non-Asian breeds [7,11,40], suggesting that the risk is not restricted to specific genetic backgrounds.

The present study has several limitations. It was conducted in vitro, involved a relatively small number of donor dogs (*n* = 5), and used ethanol extracts with unquantified concentrations of active compounds. Although this sample size was sufficient for preliminary in vitro evaluation, it limits the statistical power and generalizability of the findings. Future research including a larger number of biological replicates is warranted to confirm these results. Therefore, direct extrapolation to in vivo conditions is not possible. Nonetheless, the findings provide mechanistic evidence that fresh, dried, and especially granulated garlic can induce oxidative damage to canine erythrocytes, manifested by hemoglobin loss and Heinz body formation. Together with literature data, these results emphasize that even processed forms of garlic retain hematotoxic potential.

Future studies should focus on standardized in vivo experiments with defined doses and controlled formulations. Randomized, double-blind, placebo-controlled trials with adequate sample sizes are needed to determine both toxic thresholds and any genuine health-promoting properties of garlic in dogs. Only such evidence will allow for a balanced risk-benefit assessment and informed recommendations regarding garlic use in companion animals.

## 5. Conclusions

This in vitro study showed that garlic in all tested forms was associated with damage canine red blood cells, with dried and granulated preparations exerting stronger hemolytic effects than fresh garlic. The results suggest that processing may enhance the toxicity of garlic by increasing the reactivity of harmful compounds. While these findings cannot be directly extrapolated to in vivo conditions, they highlight the need for caution when using garlic in canine diets and call for further research to establish safe intake levels.

## Figures and Tables

**Table 1:** Comparison of commercially available garlic-containing supplements for dogs: manufacturers, countries of origin, composition, manufacturer claims, and recommended dosages. Manufacturer-provided dosage and composition information; actual content of active compounds (e.g., organosulfur compounds) not always specified.

Product	Manufacturer Claims	Composition	Recommended Dosage
GRAU Garlic Tablets (GRAU GmbH, Isselburg, Germany)	Natural protection against fleas and ticks; essential oils mask lactic acid odor; support against insect infestations; complementary feed for dogs and cats.	Garlic (*Allium sativum* L.) 95%, brewer’s yeast 5%.	Small dogs/cats: 2 tablets daily; medium dogs (≤25 kg): 3–4 tablets; large dogs (>25 kg): 5–6 tablets daily.
Canvit Garlic (Canvit, Chrášťany, Czech Republic)	Supports parasite protection (garlic, turmeric, chamomile); boosts immunity with anti-inflammatory and antioxidant effects; aids digestion and detoxification.	Lactose, dried garlic (240,000 mg/kg), lignocellulose, hydrolyzed liver, dried turmeric (5000 mg/kg), dried chamomile (2000 mg/kg), dried wild garlic (2000 mg/kg).	1 tablet per 5 kg body weight daily.
Beaphar Garlic Treats (Beaphar, Raalte, The Netherlands)	Supplement in snack form; promotes healthy skin and shiny coat; supports digestion.	Meat and animal by-products (sheep fat 95%), plant by-products (garlic 5%).	Dogs 0–20 kg: 1 treat; 20–40 kg: 2 treats; 40–60 kg: 3 treats daily.
Mikita Canvital Garlic (Mikita Canvital, Lublin, Poland)	Blend of proteins, vitamins, minerals + garlic oil; antibacterial, antiparasitic, improves appetite; recommended for active dogs, recovery, skin/coat issues.	Brewer’s yeast, sucrose, garlic oil, whey, dicalcium phosphate, calcium-magnesium carbonate, antioxidants E320/E321.	Small dogs: 1–2 tablets; medium: 2–4; large: 4–8; giant breeds: 8–12 tablets daily.
Lunderland BIO-Garlic (Lunderland Naturkost GmbH, Lüchow, Germany)	100% organic granulated garlic; regulates gut flora, aids digestion, supports immunity; antimicrobial, antiparasitic, stimulates stomach and liver.	100% garlic (granulate).	Cats/small dogs: 1 pinch daily; medium/large dogs: max ¼ tsp daily, for 6–8 weeks.
Trixie Garlic Pellets (Trixie Heimtierbedarf GmbH & Co. KG, Tarp, Germany)	Natural parasite protection; supports cardiovascular system; palatable snack form.	Cereals, plant by-products, garlic (5%), meat and animal by-products, fish, dairy, oils and fats, minerals.	Not specified by manufacturer.
Rodi Exclusive Sheep Fat with Garlic (Rodi Exclusive, Warszawa, Poland)	Supplement with sheep fat + garlic; natural parasite repellent; supports skin/coat health, appetite, energy, and digestion.	Sheep fat 97.5%, garlic 2.5%.	1 snack per 10 kg body weight daily.

**Table 2:** Percentage of erythrocytes containing Heinz bodies (%) and hemoglobin concentration (Hb, g/100 mL) in canine blood incubated with extracts of fresh, dried, and granulated garlic compared with the control group. Data are presented as mean with standard deviation (SD) and standard error of the mean (SEM). Different superscript lowercase letters within a row indicate statistically significant differences at *p* < 0.05; uppercase letters indicate highly significant differences at *p* < 0.01.

Group	Control	Fresh	Dry	Granulated	SD	SEM	*p*
Heinz bodies, %	2.27 ^Aa^	5.14 ^Aab^	10.44 ^ABbc^	14.82 ^Bc^	0.05	0.02	<0.01
SD	0.85	1.73	3.42	2.46			
SEM	0.49	1.00	1.98	1.42			
Hb, g/100 mL	15.62	16.31	12.99	12.58	2.26	0.51	0.09
SD	2.34	0.55	1.92	1.35			
SEM	1.04	0.25	0.86	0.60			

**Table 3:** Percentage of erythrocytes containing Heinz bodies (%) and hemoglobin concentration (Hb, g/100 mL) in canine blood incubated with extracts of granulated garlic at concentrations of 0.1 g/mL and 0.2 g/mL, compared with the control group. Data are presented as mean with standard deviation (SD) and standard error of the mean (SEM). Different superscript lowercase letters within a row indicate significant differences at *p* < 0.05; uppercase letters indicate highly significant differences at *p* < 0.01.

Group	Control	0.1 g/mL	0.2 g/mL	SD	SEM	*p*
Heinz bodies, %	0.30 ^A^	3.76 ^B^	7.35 ^C^	3.19	1.06	<0.01
SD	0.26	1.56	1.00			
SEM	0.15	0.90	0.58			
Hb, g/100 mL	17.54 ^a^	15.72 ^ab^	10.82 ^b^	3.62	1.21	0.093
SD	1.41	1.23	3.56			
SEM	0.81	0.271	2.05			

## Data Availability

The original contributions presented in this study are included in the article. Further inquiries can be directed to the corresponding author(s).

## Abbreviations

The following abbreviations are used in this manuscript:

AGE	Aged Garlic Extract
ANOVA	Analysis of Variance
BW	Body Weight
EDTA	Ethylenediaminetetraacetic Acid
FEDIAF	European Pet Food Industry Federation (Fédération Européenne de l’Industrie des Aliments pour Animaux Familiers)
Hb	Hemoglobin
NRC	National Research Council
PBS	Phosphate-Buffered Saline
SD	Standard Deviation
SEM	Standard Error of the Mean
VPIS	Veterinary Poisons Information Service
